# Effect of inulin-type fructans in patients undergoing cancer treatments: A systematic review

**DOI:** 10.12669/pjms.35.2.701

**Published:** 2019

**Authors:** Reihaneh Mazraeh, Fatemeh Azizi-Soleiman, Seyed Mohammad Hosein Mousavi Jazayeri, Seyyed Mohammad Ali Noori

**Affiliations:** 1 *Reihaneh Mazraeh, Nutrition and Metabolic Diseases Research Center, Ahvaz Jundishapur University of Medical Sciences, Ahvaz, Iran*; 2 *Fatemeh Azizi-Soleiman, School of Health, Arak University of Medical Sciences, Arak, Iran*; 3 *Seyed Mohammad Hosein Mousavi Jazayeri, Nutrition and Metabolic Diseases Research Center, Ahvaz Jundishapur University of Medical Sciences, Ahvaz, Iran*; 4 *Seyyed Mohammad Ali Noori, Toxicology Research Center, Ahvaz Jundishapur University of Medical Sciences, Ahvaz, Iran*

**Keywords:** Inulin, Neoplasms, Dietary Supplements, Functional Food

## Abstract

**Background and Objective:**

Current studies give us inconsistent results regarding the inulin consumption in cancer patients. The results of to-date studies are summarized in this systematic review.

**Methods:**

Web of Science (Science citation index expanded), PubMed (Medline), Embase and CENTRAL Science direct, Google scholar, Scopus and Cochrane were searched. Cochrane Collaboration’s ‘Risk of Bias’ tool was used to assess the quality of included articles.

**Results:**

Our search yielded 2652 studies after the elimination of duplicates. Three randomized controlled trials (RCTs), reporting results from 197 patients, were eligible for inclusion in the present systematic review. Risk of bias in these studies was assessed as high and moderate.

**Conclusion:**

The available evidence is inconclusive regarding the effect of inulin and oligofructose on cancer outcomes. Nonetheless, possible inulin positive effects including improved stool consistency after abdomen radiotherapy and increased stool butyrate content which is involved in controlling tumor cells proliferation and apoptosis should not be denied. Further research is needed in this area before strong conclusions can be drawn.

## INTRODUCTION

According to global cancer statistics in 2012, nearly 14.1 million new cases of cancer were diagnosed and the cancer mortality rate was 8.2 million worldwide.^1^ Globally, lung and breast cancer were the most prevalent cancers. The importance of healthy diet has been proven in cancer prevention and control, however, whether using dietary supplements during cancer treatment is effective for recovery from cancer, remains unclear.^2^ Fiber is one of the food components that has been studied a lot regarding its association with cancer. It has shown that each 5-g/d increase in fiber intake could reduce CRC-specific mortality by 18% (95% CI, 7%-28%; P = .002).^3^ Different types of fiber have different physical and chemical properties, all of which can affect digestion, appetite regulation, and energy consumption differently.^4^,^5^ In particular, there is growing interest in fermentable fibers, such as inulin-type fructans (ITFs), and the impact they have on the human body.^6^ Inulin is a carbohydrate highly available in the root of many Arsteracee such as chicory, and its hydrolysis by means of inulinase leads to a mixture of fructose and glucose.^7^,^8^ It is a naturally occurring polysaccharide composed of a mixture of oligomers and polymers containing 2 to 60 β-2-1 linked D-fructosemolecules, usually with an α-2-1 linked D-glucose end.^9^,^10^ Inulin exhibits a prebiotic effect when fermented under anaerobic conditions in the colon, preferentially stimulating the growth of bifidobacteria in the lower colon.^11^,^12^ Inulin also has potential health benefits, such as promoting immune system function, supporting the cardiovascular system, and increasing the absorption of minerals.^13^ Incorporating inulin or oligofructose in the diet of mice resulted in less tumor formation and growth as well as decreased metastases.^14^

### Description of the intervention

This review explored the impact of inulin inulin-type fructans consumption in patients undergoing cancer treatments

### Why it is important to do this review

No systematic review has been completed investigating inulin in cancer patients and the role of inulin in optimizing the management of cancer. This review will add to an overall understanding about the role of inulin in nutrition support in cancer patients. This review seeks to aid in developing an evidence-based approach to the medico-nutritional management of cancer patients, potentially justifying clinical recommendations and informing future research efforts.

## METHODS

We sought to identify relevant studies published until December 2017 using a multifaceted strategy. We searched Web of Science (Science citation index expanded), PubMed (Medline), Embase and CENTRAL, Science direct, Google scholar, Scopus and Cochrane database to identify articles fulfilling our inclusion criteria using the following search terms: cancer OR neoplasm AND inulin OR supplement OR probiotics fiber OR prebiotic. We used two recent terms to find articles conducted on inulin-type fructans. We limited the search strategy to humans. We also electronically searched the content of two leading journals in cancer, European Journal of Cancer Prevention and Journal of Clinical Gastroenterology and also searched the content of Nutrition and Cancer, British Journal of Nutrition and European Journal of Cancer to find potentially relevant abstract publications or articles. We also hand-searched the reference lists of included trials and other systematic reviews of inulin to identify additional trials. Unpublished trials were sought through trial registries (http://www.controlled-trials.com and http://www.clinicaltrials.gov). No language restriction was applied.

### Study selection

Two reviewers (RM, SMHMJ) independently screened abstract citations, retrieved articles and assessed trials for study inclusion. We included randomized controlled trials (RCTs) comparing inulin in cancer patients. Trials involving patients with any type of cancer or at any stage of cancer were eligible. No criteria were set for participant characteristics (age, gender, etc.).

### Data abstraction and assessment of methodological quality

Two reviewers (RM, SMHMJ) independently screened all articles on title/abstract. Disagreements were solved by discussion. The reference lists of these articles were screened for additional studies. Final selection for inclusion was based on the assessment of the full-text article. We abstracted information regarding the trial’s methodological quality using the Cochrane’s criteria^15^ including method of randomization, allocation concealment, blinding, incomplete outcome data (loss of follow-up). We resolved disagreement among reviewers by consensus.

**Fig.1:**
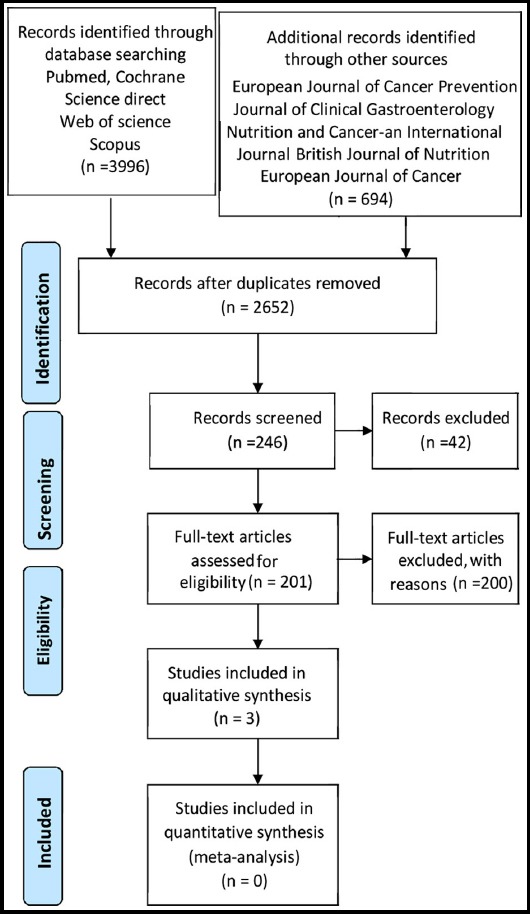
Flow diagram for identification of relevant studies.

Our search yielded 2652 records after the elimination of duplicates. Three studies^16^-^18^ were qualified for inclusion in the present systematic review. Fig.1 shows the flow diagram for identification of relevant studies.

## RESULTS

### Study identification and selection

Our search yielded 2652 records after the elimination of duplicates. Forty-two records were excluded through title and abstract screening and 204 studies were evaluated in more detail by obtaining full texts. We excluded 201 studies because they did not meet the study criteria. Three studies^16^-^18^ were qualified for inclusion in the present systematic review.

### Study description and methodological quality

A summary of each study characteristics and results is given in Table-I. Limburg et al.^17^ evaluated the effects of possible effective agents for reducing colorectal cancer risk including sulindac, atorvastatin or prebiotic. Eighty-five patients with a history of previously resected colon cancer or advanced colorectal adenomas were randomized to one of the following groups for 6 months: atorvastatin (20mg/d); sulindac 300mg/d); oligofructose-enriched inulin (12g/d); or control. Percent change in number of rectal aberrant crypt foci (ACF) (precursor of colorectal adenoma) had no significant difference within and between groups compared to baseline values or control group. Biomarkers of proliferation (Ki67) and apoptosis (caspase-3) were not different between groups at the end of intervention. However, apoptosis increased in all arms.

**Table-I:** Summary of included study characteristics.

Reference	Design	Final Subject characteristics	Treatment	Results
Marie-Christine Boutron-Ruault, et al (2009)^18^	RCT	74 Subjects with small adenomas (<10 mm in diameter), larger adenomas, or no adenomas were recruited in nine French gastroenterology departments	All subjects received a 3-mo course of 5 g of sc-FOS twice daily	Butyrate was significantly increased in the adenoma groups. cholic acid, chenodeoxycholic acid, total primary bile acids and ursodeoxycholic acid increased and fecal lithocholic acid decreased in subjects without adenoma
Paul J. Limburg et al (2011)^17^	RCT	85 patients 40 years or older with a history of previously resected colon cancer	for 6 months Arm A : Atorvastatin 20 mg qd , N = 22 Arm B : Sulindac 150 mg bid , N = 21 Arm C : ORAFTI Synergy1 6 g bid , N = 20 Arm D : Control (maltodextrin) 6 g bid , N = 22	interventions with sulindac, atorvastatin, and ORAFTI Synergy1 did not yield significant reductions in rectal ACF number, as compared with control (maltodextrin)
P Garcia-Peris et al (2015)^16^	RCT	38 female gender, diagnosis of gynecological cancer requiring postoperative pelvic RT	from one week before to three weeks after RT. The prebiotic group received a mixture of fiber (6 gr inulin and 6 gr fos) received 6 g of Malt dextrin	bowel movements per month increased decrease in the number of days with watery stools

**RT =** radiotherapy,**FOS =** fructo-oligosaccharide.

Garcia-Peris et al.^16^ conducted a 4-week randomized, double-blind, placebo-controlled trial on 38 gynecological cancer patients undergoing abdominal radiotherapy. Patients were randomly assigned to receive 12g/d of a mixture of inulin and fructo-oligosaccharide (prebiotic group) or placebo (12g/d maltodextrin). Number of days with watery stools decreased in prebiotic group (P=0.07) and increased in control group (P=0.07). At baseline, regarding quality-of-life (QOL), insomnia had the highest score in both groups. It changed to diarrhea at the end of study in placebo group, while remained unchanged in participants receiving prebiotic. However, groups did not differ in the score of QOL (overall or any of the items).

Boutron-Ruault et al.^18^ studied the effects of a 3-mo consumption of 10 g/d short-chain fructo-oligosaccharides in 74 patients with small, large or no colorectal adenomas. Biological parameters linked to colon cancer risk (short-chain fatty acids and fecal bile acids) were assessed. Mean fecal butyrate level significantly increased in those with adenoma (P=0.02). Fecal lithocholic acid significantly decreased in adenoma-free participants. However, colonic cell proliferation was not affected in any patients.

### Risk of bias in included studies

**Fig.2:**
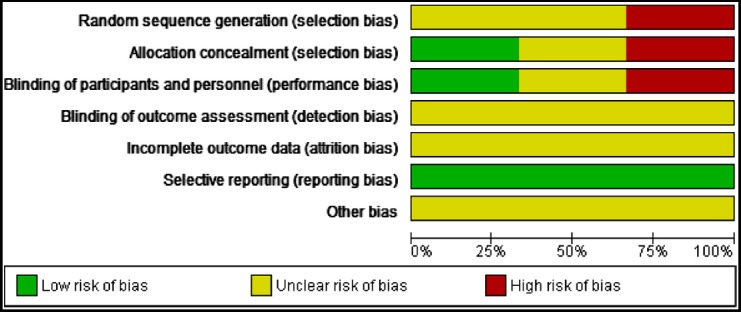
Risk of bias graph: review authors’ judgments about each risk of bias item presented as percentages across all included studies.

**Fig.3:**
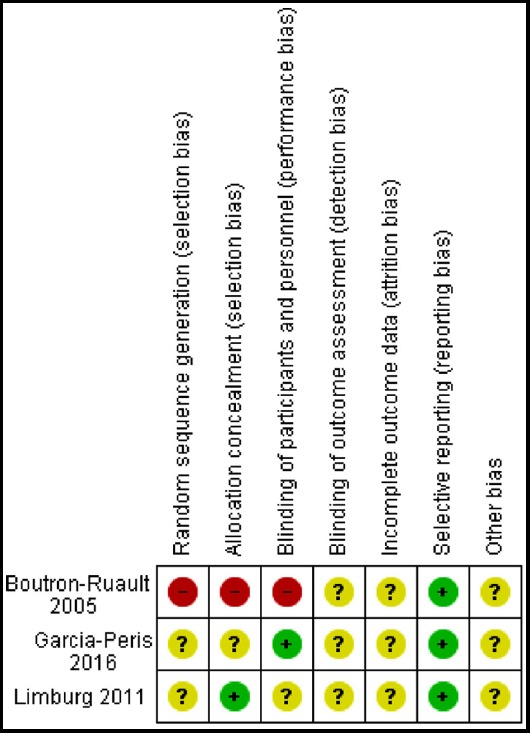
Risk of bias summary: review authors’ judgments about each risk of bias item for each included study.

The findings are presented in the ‘Risk of bias’ graph (Fig.2), which reviews the authors’ judgments about each risk of bias item shown as percentages across all included studies and the ‘Risk of bias’ summary (Fig.3), which reviews the authors’ judgments about each risk of bias item for each included study.

## DISCUSSION

Inconclusive evidence of the effects of inulin and oligofructose on cancer outcomes in humans suggests a clear need for conducting large, well-designed and controlled RCTs to reach a firm conclusion.

Most studies evaluating inulin-type oligofructose roles in cancer have been focused on CRC. Animal experiments have demonstrated that inulin and oligofructose play roles as anti-carcinogenic and anti-metastatic agents, inhibit tumor growth, and potentiate cancer treatments in colorectal cancer.^17^ Increased number of bifidobacteria in the colon^18^,^19^ and its induced cell wall preparations^20^,^21^ are involved in the cancer growth inhibition effects of inulin and oligofructose. Furthermore, proliferative and apoptotic characteristics of these two prebiotics is related to decreased availability of glucose as an essential substrate for cancer cells.^22^-^25^ Additionally, in a review by Pool-Zobel^26^ on beneficial effects of inulin-type fructans in colorectal cancer, the proposed mechanisms were as follows: 1) decreasing exposure to genotoxic carcinogens in the gut or their genotoxic impacts; 2) growth inhibition; 3) gene expression modulation; and 4) lowering metastasis activities of colon tumor cells. Nevertheless, few studies on other types of cancer have shown that these non-digestible carbohydrates produce their anti-cancer effects by lowering serum glucose and fatty acids concentration required for cancer cells growth.

Two of three studies included in our review were conducted in colorectal cancer patients; one had null effects and the other showed an increase in fecal butyrate concentration after prebiotic supplementation. Supplementation of healthy adults who underwent colonoscopy with a mixture of inulin and oligofructose did not change cell proliferation;^27^ however, some limitations should be considered when interpreting the results. As authors pointed disagreements between endoscopic diagnosis of ACF and histologic evaluation, possible greater impacts of symbiotic vs. probiotics alone, and measuring biomarkers from rectum only could affect the observed null results. It has been postulated that protective properties of butyrate against colon cancer is related to inhibiting proliferation, inducing differentiation and apoptosis in transformed cells including cancer cells.^28^ Although butyrate increased in cancer patients in Boutron-Ruault study, cell proliferation remained unchanged. Perhaps if Limburg et al. assessed apoptosis and number of ACF, they might report similar results.

Advantages of inulin in the prevention of radiation enteritis in gynecologic cancer patients are consequences of intestinal flora recovery. Most studies in this area are limited to probiotics.^29^,^30^ Applying probiotics in patients with pelvic cancer reduce radiation-induced diarrhea in patients with pelvic cancer.^30^ It seems that the results are dependent on type of probiotics and the dose used.^16^

### Limitations of the study

Several points should be considered while interpreting our results. Short study duration, small sample size, different dose of prebiotics, recruiting different patients, dissimilar biomarkers, and non-application of combination of biomarkers^31^ are propounded as presumable causes of finding inconsistent results. There are also some studies in mice models in which using inulin-type fructans has been resulted in enhanced proliferation of adenomas.^32^-^34^ Therefore, more studies are required to find real effects in humans.

## CONCLUSION

No definite conclusion could be drawn. Nevertheless, possible positive effects of inulin and oligofructose should not be neglected. These effects include improved stool consistency after abdomen radiotherapy and increased stool butyrate content which is involved in controlling tumor cells proliferation and apoptosis. Future large, well-designed randomized controlled trials should be designed in order to discover inulin and oligofructose efficiency in controlling cancer outcomes by virtue of various appropriate biomarkers.
